# Adverse childhood experiences and psychological functioning among women with schizophrenia or bipolar disorder: population-based study

**DOI:** 10.1192/bjp.2023.128

**Published:** 2024-01

**Authors:** Ole Köhler-Forsberg, Fenfen Ge, Arna Hauksdóttir, Edda Bjork Thordardottir, Kristjana Ásbjörnsdóttir, Harpa Rúnarsdóttir, Gunnar Tómasson, Jóhanna Jakobsdóttir, Berglind Guðmundsdóttir, Andri Steinþór Björnsson, Engilbert Sigurðsson, Thor Aspelund, Unnur A. Valdimarsdottir

**Affiliations:** Psychosis Research Unit, Aarhus University Hospital – Psychiatry, Denmark; and Department of Clinical Medicine, Aarhus University, Denmark; Centre of Public Health Sciences, Faculty of Medicine, University of Iceland, Iceland; Centre of Public Health Sciences, Faculty of Medicine, University of Iceland, Iceland; and Mental Health Services, Landspitali, The National University Hospital of Iceland, Iceland; Faculty of Medicine, University of Iceland, Iceland; and Mental Health Services, Landspitali, The National University Hospital of Iceland, Iceland; Faculty of Psychology, University of Iceland, Iceland; Faculty of Medicine, University of Iceland, Iceland; Centre of Public Health Sciences, Faculty of Medicine, University of Iceland, Iceland; Unit of Integrative Epidemiology, Institute of Environmental Medicine, Karolinska Institute, Sweden; and Harvard T.H. Chan School of Public Health, USA

**Keywords:** Adverse childhood experiences, childhood deprivation, schizophrenia, bipolar disorder

## Abstract

**Background:**

Adverse childhood experiences (ACEs) are well-known risk factors for schizophrenia and bipolar disorder.

**Aims:**

The aim was to study the associations between specific ACEs and psychological functioning in women with schizophrenia or bipolar disorder.

**Method:**

Among 29 367 women (mean age 44 years) from the Icelandic Stress-And-Gene-Analysis (SAGA) study, 534 (1.8%, mean age 40) reported having been diagnosed with schizophrenia or bipolar disorder, which were combined to ‘severe mental disorders’. Participants reported on 13 types of ACEs, childhood deprivation and psychological functioning (defined as coping ability and current symptoms of depression, anxiety and sleep disturbances). Adjusted Poisson regression calculated prevalence ratios (PRs) between ACEs and severe mental disorders. Linear regression assessed the association between ACEs and psychological functioning among women with a severe mental disorder.

**Results:**

Women with a severe mental disorder reported more ACEs (mean 4.57, s.d. = 2.82) than women without (mean 2.51, s.d. = 2.34) in a dose-dependent manner (fully-adjusted PR = 1.23 per ACE, 95% CI 1.20–1.27). After mutual adjustment for other ACEs, emotional abuse, sexual abuse, mental illness of a household member, emotional neglect, bullying and collective violence were associated with severe mental disorders. Among women with severe mental disorders, a higher number of ACEs was associated with increased symptom burden of depression (β = 2.79, 95% CI = 1.19–4.38) and anxiety (β = 2.04, 95% CI = 0.99–3.09) including poorer sleep quality (β = 0.83, 95% CI = 0.07–1.59). Findings were similar for schizophrenia and bipolar disorder separately.

**Conclusion:**

Women with schizophrenia or bipolar disorder show a strong history of ACEs, which may interfere with their psychological functioning and, therefore, need to be addressed as part of their treatment, for example, with trauma-focused psychotherapy.

## Background

Schizophrenia and bipolar disorder are severe mental disorders resulting in a high burden and immense costs for the society.^1^ These disorders represent multifactorial complex lifelong challenges for the patients and their families, and may result in multiple severe episodes and additional comorbid symptoms throughout life, such as depression, anxiety and sleeping problems.^1^ Adverse childhood experiences (ACEs) are well-established environmental risk factors for these disorders^2–7^ with a recent meta-umbrella systematic review on modifiable risk factors showing that the largest global population attributable fraction (PAF) of schizophrenia spectrum disorders was linked to childhood adversities (PAF 38%),^6^ indicating a large preventive capacity. As detailed knowledge on specific ACEs represents important information to guide preventive efforts, it was of particular interest that childhood sexual abuse showed a 13% PAF for the development of depression.^6^ However, less is known about the extent to which specific ACEs are associated with schizophrenia and bipolar disorder. A recent study emphasised that specific ACEs may differentially predict specific mental disorders,^8^ but most previous studies were often limited by information on only a few specific ACEs,^9^ small heterogeneous cohorts or no representative reference group.^6,7^ This emphasises the need for large trans-diagnostic studies investigating the effect of a broad range of ACEs compared with a representative reference group.

Furthermore, among individuals with schizophrenia or bipolar disorder, ACEs correlate with a worse clinical course and greater severity of psychotic symptoms and suicidal ideation.^3,4,10^ Psychotic and manic symptoms represent the diagnostic criteria for schizophrenia and bipolar disorder, respectively, but symptoms of depression and anxiety including sleep disturbances have a large impact on everyday functioning. Resilience represents an important parameter to cope with symptoms of severe mental disorders as well as the challenges of everyday life. Yet, whether ACEs are associated with a negative impact on these features of general psychological functioning among individuals living with a schizophrenia or bipolar disorder diagnosis has not been sufficiently studied.

## Aims

Using a large nationwide representative Icelandic sample of women from the general population, we therefore aimed to study the following research questions.

- Are specific types of ACEs differentially associated with schizophrenia or bipolar disorder than other ACEs?
- Among women with schizophrenia or bipolar disorder, is exposure to multiple ACEs associated with suboptimal psychological functioning, indicated by a higher symptom burden of depression, anxiety, sleep disturbances and lower coping ability?

## Method

### Stress-And-Gene-Analysis cohort about Icelandic women

**Fig. 1:**
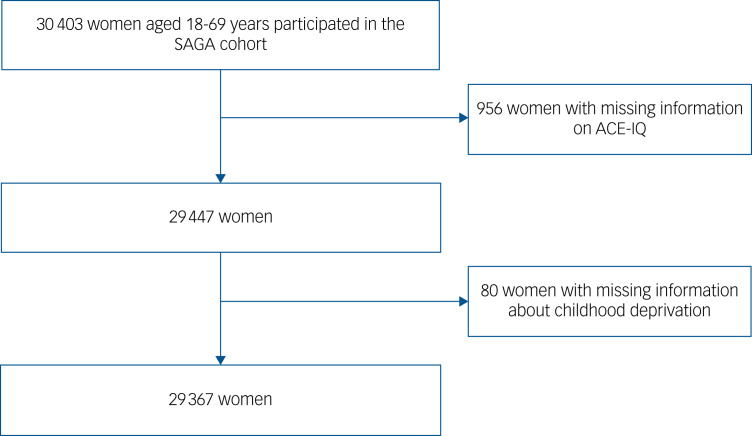
The flow chart of study population. ACE-IQ, Adverse Childhood Experience International Questionnaire; SAGA, Stress-And-Gene-Analysis.

The Stress-And-Gene-Analysis (SAGA) cohort is a study of women aged 18–69 years residing in Iceland in 2018.^11–13^ All Icelandic-speaking women with an address or telephone number (approximate *n*  104 197) were invited to participate in the study between March 2018 and July 2019. The participants of the SAGA cohort answered an extensive web-based questionnaire including questions on for example, demographic factors, psychological trauma history (in childhood and adulthood), psychological health and history of various diseases. As shown in a previous study about the SAGA cohort, the participants represent the general population of Icelandic women in terms of age, education, geographic location of residence and monthly wages.^12^ A total of 29 367 women (mean age 44 years) with sufficient information on ACEs and childhood deprivation were included in the present study (Fig. 1). All participants signed an informed consent. The authors assert that all procedures contributing to this work comply with the ethical standards of the relevant national and institutional committees on human experimentation and with the Helsinki Declaration of 1975, as revised in 2008. All procedures involving patients were approved by the National Bioethics Committee of Iceland (reference number: 17–238) and the Icelandic Data Protection Authority.

### Information on schizophrenia and bipolar disorder diagnoses

Women reported whether they had ever been diagnosed by a healthcare professional (e.g. a psychologist or medical doctor) with either schizophrenia or bipolar disorder. A total of 108 women (0.4%) reported a diagnosis of schizophrenia and 479 (1.6%) of bipolar disorder, with 53 of these women reporting being diagnosed with both disorders, resulting in 534 women with schizophrenia or bipolar disorder. We had no information on the date of the diagnosis nor specific diagnostic details. Self-report information on severe mental disorders has been shown to represent valid information relevant for research purposes.^14^ For the overall analyses, we grouped women with schizophrenia or bipolar disorder together as having severe mental disorders. Women who did not report a schizophrenia or bipolar disorder diagnosis are referred to as the reference group.

### Measurement of ACEs and childhood deprivation

ACEs were measured with a modified version of the ACE International Questionnaire (ACE-IQ) developed by the World Health Organization.^15^ The ACE-IQ consists of 39 items assessing the frequency of exposure to 13 types of ACEs before the age of 18 years: emotional neglect, physical neglect, emotional abuse, physical abuse (the latter four items were referring specifically to abuse/neglect by parents or a guardian), sexual abuse, domestic violence, living with a household member who misuses drugs and/or alcohol, living with a household member who is mentally ill or suicidal, incarceration of a household member, parental death or separation/divorce, being bullied, witnessing community violence and exposure to war/collective violence. Response options varied between items and could either be answered on a five-point scale (ranging from 0 (never) to 4 (always)), on a four-point scale (from 0 (never) to 3 (many times)) or answered dichotomously 0 (no) and 1 (yes). In the main analysis, the response option ‘cannot/will not answer’ was treated as no childhood adversity for that respective question. In the present study, we followed the recommended frequency scoring system, which takes into account the level of exposure for each ACE. We then generated three types of exposure variables:

- a continuous ACE-IQ total score ranging from 0 to 13, reflecting the number of ACEs the participants were exposed to;
- the total score categorised based on the distribution of the sample (0–2, 3–4 and ≥5 ACEs); and
- binary variables for each individual ACE type, coded as 0 (unexposed) and 1 (exposed).

Childhood deprivation was assessed with the question: ‘Was your family's economic situation ever so bad that you suffered any deprivation as a consequence? For example, this could apply to deprivation of nutritious food and/or deprivation of warm clothes and appropriate footwear during the winter months’, with response options ranging from 0 (never) to 3 (often). For analytical purposes, we grouped childhood deprivation into ‘no’ (never and rarely occurring) and ‘yes’ (sometimes and often), as has been done previously.^12^

### Measurements of psychological functioning

Questions on psychological functioning covered present-state mental symptoms and coping ability. Women reported their symptoms via the following measures, with higher scores indicating greater symptom severity: depressive symptoms were assessed with the nine-item Patient Health Questionnaire (PHQ-9) with a score ≥10 representing a well-established validated cut-off for a probable diagnosis of depression.^16^ Symptoms of anxiety were assessed with the Generalized Anxiety Disorder 7-item scale (GAD-7) with a score ≥10 representing a well-established validated cut-off for clinically relevant anxiety symptoms.^17^ Sleep disturbances were assessed with the Pittsburgh Sleep Quality Index (PSQI), with 19 individual items generating seven component scores and with a PSQI ≥6 indicating clinically significant sleep disturbances.^18^

We imputed data for women who responded to more than 75% of items on the PHQ-9, GAD-7 and PSQI. Mean scores were used to replace missing values in both PHQ-9 and GAD-7 questionnaires. For the PSQI questionnaire, we replaced missing values with single imputation using predictive mean matching (PMM, with default *d* = 5 and 20 iterations).^19^

Perceived coping ability was assessed with the 10-item version of the Connor–Davidson Resilience scale (CD-RISC-10),^20^ measuring individuals’ perceptions of their ability to cope effectively with stress and adversity. The CD-RISC-10 scale has demonstrated good reliability and validity.^20^ Items were summed to create a total score ranging from 0 to 40 with a higher score indicating higher levels of perceived coping ability.

### Covariates

We included the following covariates: age at responding; civil status (divided into married or in a relationship and single or widowed); educational level categorised as primary education, secondary education (high school or vocational education), tertiary education A (BSc or equivalent) and tertiary education B (MSc or above); current personal monthly income (categorised into low (≤US$2527/month), moderate (US$2528 to US$5897/month), high (>US$5898); conversion rates according to Central Bank of Iceland, 17 October 2018); body mass index (BMI); and smoking status (never, previous and current).

### Statistical analysis

We compared the study population's characteristics between women with and without severe mental disorders using the *t*-test for age (as continuous variables) and the chi^2^-test for other variables and determined correlations between specific types of ACEs via rank-order correlations.

For the primary analyses, we grouped women with schizophrenia or bipolar disorder as one group, i.e. women with a severe mental disorder. We estimated prevalence ratios (PRs) including 95% CI of a continuous ACE-IQ total score via Poisson log-binomial regression models with the sandwich variance estimators.^21^ We also compared women who endorsed different numbers of ACEs (3–4 and ≥5 ACEs) with women with 0–2 ACEs and women with childhood deprivation to women without childhood deprivation. We used the group of women with 0–2 ACEs as the comparison group because the majority of women (79.7% among women without a severe mental disorder *v.* 95.7% among women with a severe mental disorders) reported at least one ACE. We conducted a model adjusted for age and a model additionally adjusted for all other covariates.

Next, we estimated the associations of type-specific ACEs and severe mental disorders. To determine the independent associations of type-specific ACEs and severe mental disorders, we first ran type-specific analyses separately adjusted for all covariates, then re-ran the analysis additionally adjusted for other types of ACEs.^22^

Finally, we performed analyses specifically among women with a severe mental disorder studying the association of ACEs (0–2, 3–4 and ≥5 ACEs) and childhood deprivation with psychological functioning (i.e. the severity of symptoms of depression, anxiety, sleep disturbances and resilience). We used both linear regression and Poisson log-binomial regression models to calculate the beta coefficient and PR adjusted for all covariates, respectively.

We performed several sensitivity and subgroup analyses. First, we re-ran all analysis separately for women with schizophrenia or bipolar disorder, respectively. Second, to assess the effect of missingness on our results, we re-ran our analysis restricted to participants without the ‘cannot/will not answer’ option on the ACE-IQ. Third, we replaced ‘cannot/will not answer’ with single imputation using PMM (with default *d* = 5 and 20 iterations).^19^ Fourth, as divorce is frequently occurring in Nordic societies and might not necessarily represent a severe ACE, we performed all analyses excluding this variable as an ACE. Fifth, as women with schizophrenia or bipolar disorder were younger and since recall bias might increase with age, we performed the analyses stratified by age.

## Results

### Characteristics of the study population

Compared with the reference group, women reporting a diagnosis of severe mental disorder were younger, more often single/widowed, less educated, more often smokers and had lower income, higher BMI, greater severity of depressive, anxiety and sleeping symptoms, and had lower coping ability (Table 1). Similar findings were noted for schizophrenia and bipolar disorder separately (Supplementary Table 1 available at https://doi.org/10.1192/bjp.2023.128).

**Table 1:** Characteristics of study population

Characteristic	No severe mental disorder group	Severe mental disorder group	Overall
*N*	28 833	534	29 367
Age^a^, years: mean (s.d.)	43.7 (13.7)	40.1 (12.9)	43.7 (13.7)
Age^a^ group, years: *n* (%)			
18–29	5649 (19.6)	140 (26.2)	5789 (19.7)
30–39	5895 (20.4)	138 (25.8)	6033 (20.5)
40–49	6371 (22.1)	111 (20.8)	6482 (22.1)
50–59	6532 (22.7)	97 (18.2)	6629 (22.6)
60–69	4386 (15.2)	48 (9.0)	4434 (15.1)
Civil status,^a^ *n* (%)			
Married or in a relationship	21 814 (75.7)	328 (61.4)	22 142 (75.4)
Single or widowed	6869 (23.8)	200 (37.5)	7069 (24.1)
Missing data	150 (0.5)	6 (1.1)	156 (0.5)
Highest education,^a^ *n* (%)			
Primary	4189 (14.5)	125 (23.4)	4314 (14.7)
Secondary	8945 (31.0)	197 (36.9)	9142 (31.1)
TertiaryA	9115 (31.6)	145 (27.2)	9260 (31.5)
TertiaryB	6469 (22.4)	62 (11.6)	6531 (22.2)
Missing data	115 (0.4)	5 (0.9)	120 (0.4)
Current personal monthly income,^a^^,^^b^ *n* (%)			
Low	8532 (29.6)	287 (53.7)	8819 (30.0)
Moderate	15 279 (53.0)	209 (39.1)	15 488 (52.7)
High	3871 (13.4)	18 (3.4)	3889 (13.2)
Missing data	1151 (4.0)	20 (3.7)	1171 (4.0)
BMI,^a^^,^^c^ *n* (%)			
Underweight	267 (0.9)	12 (2.2)	279 (1.0)
Normal	9448 (32.8)	119 (22.3)	9567 (32.6)
Overweight	8505 (29.5)	170 (31.8)	8675 (29.5)
Obesity	8002 (27.8)	221 (41.4)	8223 (28.0)
Unknown	2611 (9.1)	12 (2.2)	2623 (8.9)
Smoking status,^a^ *n* (%)			
Never	12 612 (43.7)	148 (27.7)	12 760 (43.5)
Previous	9627 (33.4)	200 (37.5)	9827 (33.5)
Current	3968 (13.8)	182 (34.1)	4150 (14.1)
Unknown	2626 (9.1)	4 (0.7)	2630 (9.0)
CD-RISC-10, mean (s.d.)^a^^,^^d^	27.4 (7.46)	20.6 (8.72)	27.30 (7.54)
GAD-7, mean (s.d.)^a^^,^^e^	6.36 (4.89)	11.1 (5.78)	6.45 (4.95)
PHQ-9, mean (s.d.)^a^^,^^f^	7.38 (5.92)	14.3 (7.09)	7.52 (6.02)
PSQI, mean (s.d.)^a^^,^^g^	7.59 (4.00)	10.7 (4.23)	7.64 (4.03)

BMI, Body Mass Index; CD-RISC-10, 10-item version of the Connor-Davidson Resilience scale; GAD-7: 7-item Generalized Anxiety Disorder; PHQ-9, 9-item Patient Health Questionnaire; PSQI, Pittsburgh Sleep Quality Index.

a. *P* < 0.001, i.e., the women with versus without a severe mental disorder differed significantly on all covariates.

b. Low: ≤US$2527/month; medium: US$2528 to US$5897/month; high: US$5898/month.

c. Underweight: BMI < 18.5; normal weight: BMI 18.5–24.9; overweight: BMI 25.0–29.9; obesity: BMI ≥ 30.0.

d. Coping ability was assessed with the 10-item version of the CD-RISC-10.

e. Past 2-week anxiety symptoms were assessed with the 7-item GAD-7.

f. Past 2-week depressive symptoms were assessed with the 9-item PHQ-9.

g. Past month sleep disturbances were assessed with the 19-items PSQI.

### Associations of ACEs and childhood deprivation with severe mental disorders

Compared with the reference group, women with a history of severe mental disorders had higher ACE scores (mean 4.57, s.d. = 2.82, *v*. mean 2.51, s.d. = 2.34), reported ACEs more frequently (48% reported ≥5 ACEs *v.* 19%) and experienced more childhood deprivation (27% *v.* 13%). ACEs and childhood deprivation were associated with higher PRs for having a severe mental disorder (Supplementary Table 2). Higher number of ACEs was associated with increasing PRs for having a severe mental disorder (fully adjusted PR = 1.23 per ACE; 95% CI 1.20–1.27) showing a dose–response association. Specifically, women who experienced 3–4 ACEs and ≥5 ACEs had a PR of 1.81 (95% CI 1.44–2.27) and 3.54 (95% CI 2.92–4.28), respectively (Supplementary Table 2). The result pattern was largely similar for schizophrenia or bipolar disorder separately (Supplementary Table 2), when stratifying for median age (Supplementary Table 3), when excluding parental divorce as an ACE (Supplementary Table 4), and when restricting this to ‘complete cases’ (Supplementary Table 5), although the 95% CIs were wide in the latter analyses, potentially owing to the small sample size. PMM analyses to assess the impact of missing data showed similar results (Supplementary Table 6).

**Fig. 2:**
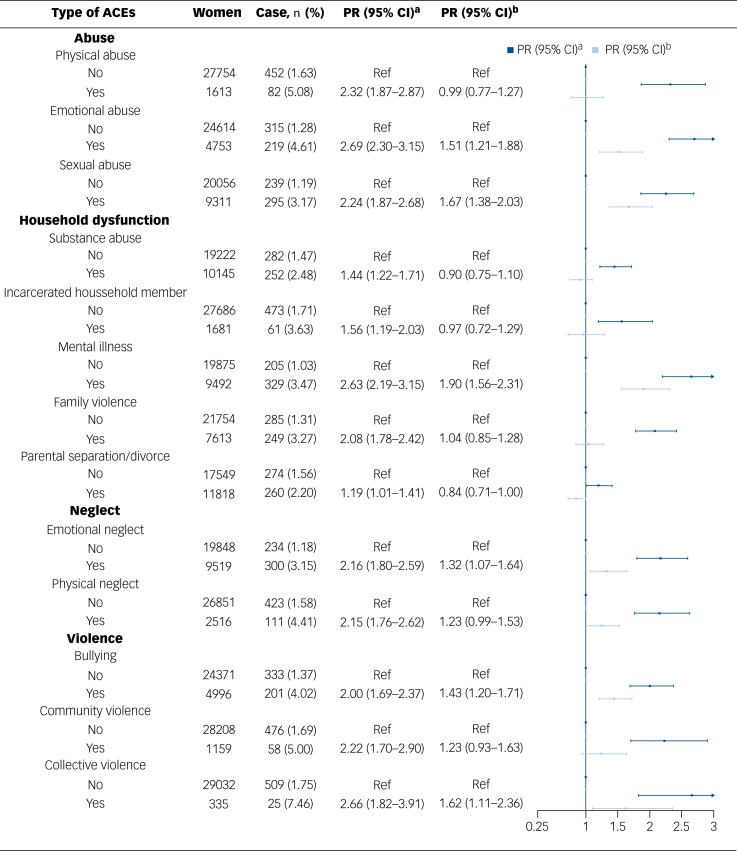
Associations between the types of adverse childhood experience (ACEs) and the prevalence of severe mental disorder. ‘case’ refers to women with a severe mental disorder. ^a^Adjusted for age, highest education, civil status, current personal monthly income, smoking status and body mass index. ^b^Additionally adjusted for other types of ACEs. PR = prevalence ratio.

The 13 specific types of ACEs were weakly to moderately correlated with each other (ranging from 0.05 to 0.51) with emotional abuse having the strongest correlation with other ACEs (Supplementary Fig. 1). Regarding the specific ACEs, all ACEs correlated with higher PRs for history of severe mental disorders when not including mutual adjustment for other ACEs, with the PRs differing between the specific ACEs (Fig. 2). When including mutual adjustment for all other ACEs, the following ACEs were associated with higher PRs for having a severe mental disorder: emotional abuse (PR = 1.51, 95% CI = 1.21–1.88), sexual abuse (PR = 1.67, 95% CI = 1.38–2.03), mental illness of a household member (PR = 1.90, 95% CI = 1.56–2.31), emotional neglect (PR = 1.32, 95% CI = 1.07–1.64), bullying (PR = 1.43, 95% CI = 1.20–1.71) and collective violence (PR = 1.62, 95% CI = 1.11–2.36). Similar findings were noted for schizophrenia and bipolar disorder separately, whereas the association with emotional and sexual abuse was more pronounced in schizophrenia than in bipolar disorder (Supplementary Table 7).

### Association between ACEs and current symptomatology

When specifically studying women with a severe mental disorder, we found that ACEs were associated with a greater current symptom burden of depression and anxiety including poorer sleeping quality, whereas childhood deprivation was associated with greater symptom burden of depression and anxiety (Table 2). We observed similar patterns for specific types of ACEs (Supplementary Table 8). The severity of depression, anxiety and sleep disturbances increased (indicated by higher β-values) and coping ability decreased (indicated by a lower β-value) among women with a severe mental disorder and higher number of ACEs. Compared with women with 0–2 ACEs, women who reported ≥5 ACEs were more likely to have symptoms of anxiety (PR = 1.29, 95% CI 1.08–1.55), depression (PR = 1.27, 95% CI 1.18–1.36) and sleep disturbances (PR = 1.10, 95% CI 1.00–1.21, Supplementary Table 9).

**Table 2:** Linear regression to assess the association between adverse childhood experiences (ACEs) and psychological functioning among women with a severe mental disorder (*n* = 534)

Characteristic	Women	Anxiety,^a^	Depression,^b^	Sleep,^c^	Coping ability,^d^
		β (95% CI)^e^	β (95% CI)^e^	β (95% CI)^e^	β (95% CI)^e^
Number of ACEs					
0–2 ACEs	155	Reference	Reference	Reference	Reference
3–4 ACEs	122	0.61 (−0.34 to 1.57)	0.51 (–0.83 to 1.84)	−0.06 (−0.81 to 0.69)	−0.37 (−2.75 to 2.01)
≥5 ACEs	257	2.04 (0.99 to 3.09)	2.79 (1.19 to 4.38)	0.83 (0.07 to 1.59)	−1.04 (−3.80 to 1.73)
Childhood deprivation					
No	392	Reference	Reference	Reference	Reference
Yes	142	0.63 (0.45 to 0.80)	1.63 (0.63 to 2.64)	0.23 (−0.21 to 0.66)	−0.76 (−1.71 to 0.19)

BMI, body mass index.

a. Anxiety symptoms were assessed with the 7-item Generalized Anxiety Disorder (GAD-7), with higher scores indicating greater symptom severity.

b. Depressive symptoms were assessed with the 9-item Patient Health Questionnaire (PHQ)-9, with higher scores indicating greater symptom severity.

c. Sleep disruptions were assessed with the 19-items Pittsburgh Sleep Quality Index (PSQI), with higher scores indicating greater symptom severity.

d. Coping ability was assessed with the 10-item Connor-Davidson Resilience scale (CD-RISC-10), with lower scores indicating worse resilience.

e. Adjusted for age, highest education, civil status, current personal monthly income, smoking status and BMI.

## Discussion

### Main findings

Based on the large and nationwide representative sample of Icelandic women, the present study comprehensively demonstrates a dose–response association between ACEs and severe mental disorders. Of 13 studied ACEs, we found seven to be associated with schizophrenia or bipolar disorder, the strongest associations were with mental illness of a household member and emotional and sexual abuse, but also emotional neglect, bullying and collective violence. Finally, and with important implications for clinical management, we found that among women with schizophrenia or bipolar disorder, the presence of ACEs was strongly associated with worse psychological functioning.

### Comparison with findings from previous studies

Previous research on ACEs has primarily focused on ACEs as a risk factor for developing mental disorders and hence representing a potential target for prevention.^6^ The detailed assessment of specific ACEs in the present study indicates a pattern of some specific ACEs being more strongly associated with severe mental disorders than others, as well as potentially different roles of some specific ACEs for different mental disorders. In line with our findings, a recent umbrella review^6^ found that childhood sexual abuse was particularly strongly correlated with risk of developing depression, although such a fine-grained evaluation on the potential role of other ACEs was not possible because of missing data on specific ACEs in individual mental disorders.^3,6,7^ Another recent study suggested potential differences between specific ACEs and the risk for specific mental disorders.^8^ The present findings suggest that sexual but also emotional abuse is associated with a higher prevalence of both schizophrenia and bipolar disorder. Severe physical abuse has also been linked to subsequent risk of a mental disorder.^23^ Although we cannot draw firm conclusions because of low statistical power in the analyses of specific types of ACEs, it seems that there exist important differences between specific ACEs and their associations with severe mental disorders that need to be considered in the context of prevention of these disorders. Furthermore, bullying and collective violence represent ACEs that can be targeted preventively, with bullying particularly representing a frequently occurring traumatic experience that seems highly relevant to target by preventive approaches. In addition, mental illness of a family member was considerably more frequent among women with schizophrenia but also bipolar disorder compared with the reference group. Although genetic factors may play a role, the negative impact of growing up with family members with severe mental disorders is well described^24^ and the present results further emphasise the importance of supporting children of individuals with severe mental disorders with the aim of preventing the development of mental disorders.^24,25^

Importantly, our study adds new knowledge on the role of ACEs for general psychological functioning among individuals with severe mental disorders. Our findings indicate a strong negative impact on a large variety of mental health symptoms affecting the everyday functioning of these individuals, thus extending previous studies indicating that ACEs are associated with a worse clinical course.^3,4,10^ Hence, ACEs should represent an important treatment focus among individuals with schizophrenia or bipolar disorder, for example, via trauma-focused psychological interventions such as trauma-based psychotherapy, because of their detrimental effect on psychological functioning.

Potential explanations for the observed associations include psychological and biological models with these potentially being intercorrelated. The psychological stress of ACEs can persist into adulthood, thereby affecting adult mental health.^26^ Studies have shown that childhood adversities invoke stress-induced neurodevelopmental changes,^26^ such as hypothalamic–pituitary axis dysregulation and chronically elevated stress hormone levels causing structural brain changes, which can increase the risk for psychopathology. Furthermore, ACEs lead to aberrant DNA methylation and thus important epigenetic modifications, with these environmental imprints on the human epigenome persisting into adulthood.^27^ It is plausible that these mechanisms affect the individual during the entire life course and hence have a negative impact on psychological symptoms beyond representing a risk factor for developing a mental disorder.

### Strengths and limitations

The strengths of the present study include the detailed assessment of a wide range of ACEs and childhood deprivation with detailed information on several important covariates, and the large and representative study population that was specifically designed to study the association between life course trauma and subsequent mental health in women.

Our study has to be interpreted in the light of potential limitations. First, our study is cross-sectional, thus it is not possible to draw any conclusions on the directionality of the observed associations. Second, women reported on lifetime diagnoses of schizophrenia or bipolar disorder and were not interviewed with a clinical diagnostic interview for participation in the present study. In addition, we had no information on the onset of symptoms or the date of the diagnoses. Nevertheless, schizophrenia and bipolar disorder represent severe mental disorders with good validity of self-reported diagnoses^14^ and with these disorders having a lifelong impact on affected individuals. Third, we had no information on the diagnostic symptoms for schizophrenia and bipolar disorder (i.e. psychotic or manic) nor on the severity of the disorder or psychopharmacological treatment. Fourth, all variables represent self-reported measures, and although this is a well-established approach for assessing ACEs, we cannot exclude the possibility that diagnoses of severe mental disorders or current mental health symptoms affect retrospective reports of ACEs. Fifth, the present study only included women. Sixth, the relatively higher rate of bipolar disorder (1.6%) than schizophrenia (0.4%) may be because individuals with schizophrenia often experience a chronic course, which may have resulted in some women with schizophrenia not participating in this study.

### Implications

The findings of the present study strengthen and extend previous findings on the detrimental impact of ACEs in women diagnosed with schizophrenia or bipolar disorder. Specific ACEs, including mental illness of a household member and sexual and psychological abuse, are strongly associated with these severe disorders, indicating the importance of considering specific ACEs in preventive approaches. Furthermore, ACEs and childhood deprivation were associated with worse psychological functioning among women with severe mental disorders. Our findings therefore strongly suggest that identifying a history of ACEs is important in the assessment and subsequent treatment of women with schizophrenia and bipolar disorder.

## Supporting information

Köhler-Forsberg et al. supplementary material Köhler-Forsberg et al. supplementary material

## Data Availability

The data used in this study are compiled from the Stress-And-Gene-Analysis (SAGA) cohort. We cannot make the data publicly available because of Icelandic laws regarding data protection and the approval for the current study granted by the National Bioethics Committee (NBC) of Iceland. The SAGA cohort contains sensitive data and all use of data are restricted to scientific purposes only subjected to approval of the NBC (email: vsn@vsn.is). Interested researchers can obtain access to deidentified data by submitting a proposal to the SAGA cohort data management board (email: afallasaga@hi.is) which assists with submitting an amendment to the NBC.
