# Supplementary material for: Adverse childhood experiences and psychological functioning among women with schizophrenia or bipolar disorder: population-based study
Source: Br J Psychiatry. 2024 Jan;224(1):6–12. doi: 10.1192/bjp.2023.128 (PMC10751941; doi:10.1192/bjp.2023.128)
Supplement: Köhler-Forsberg et al. supplementary material [file S0007125023001289sup001.docx]

**Supplementary material for**

**Adverse childhood experiences and psychological functioning among women with schizophrenia or bipolar disorder: A population-based study**

Ole Köhler-Forsberg*^1,2^, Fenfen Ge*^3^, Arna Hauksdóttir^3^, Edda Bjork Thordardottir^3,4^, Kristjana Ásbjörnsdóttir^3^, Harpa Rúnarsdóttir^3^, Gunnar Tómasson^3^, Jóhanna Jakobsdóttir^3^, Berglind Guðmundsdóttir^5^, Andri Steinþór Björnsson^6^, Engilbert Sigurðsson^5^, Thor Aspelund^3^, Unnur A. Valdimarsdottir^3,7,8^

Table of Contents

[Supplementary table 1 Characteristics of the women with schizophrenia and bipolar disorder 2](#_Toc139379064)

[Supplementary table 2 Associations between the number of ACEs and the prevalence of A) a severe mental disorder, B) schizophrenia or C) bipolar disorder separately. 3](#_Toc139379065)

[Supplementary table 3 Associations between the number of ACEs and the prevalence of severe mental disorder stratified by median age 4](#_Toc139379066)

[Supplementary table 4 Associations between the number of ACEs and the prevalence of severe mental disorder (excluding parental divorce/separation) 5](#_Toc139379067)

[Supplementary table 5 Associations between the number of ACEs and the prevalence of severe mental disorder (complete cases) 6](#_Toc139379068)

[Supplementary table 6 Associations between the number of ACEs and the prevalence of severe mental disorder (single imputation using method predictive mean matching to impute ACE-IQ) 7](#_Toc139379069)

[Supplementary table 7 Associations between the types of ACEs and the prevalence of bipolar disorder or schizophrenia 8](#_Toc139379070)

[Supplementary table 8 Linear regression to assess the association between types of ACE and psychological functioning among women with severe psychiatric disorders (n= 534) 10](#_Toc139379071)

[Supplementary table 9 Poisson regression to assess the association between ACEs and psychological functioning among women with severe mental disorder (n= 534) 12](#_Toc139379072)

[Supplementary Figure 1 Rank order correlations for ACE subtypes 13](#_Toc139379073)

## **Supplementary table 1** Characteristics of the women with schizophrenia and bipolar disorder

|  | Schizophrenia | Bipolar disorder |
| --- | --- | --- |
|  | 108 | 479 |
| Age, mean (SD) | 39.05(14.3) | 40.43(12.9) |
| Age group (%) |  |  |
| 18-29 | 36(33.3) | 120(25.1) |
| 30-39 | 24(22.2) | 125(26.1) |
| 40-49 | 17(15.7) | 101(21.1) |
| 50-59 | 19(17.6) | 88(18.4) |
| 60-69 | 12(11.1) | 45(9.4) |
| Civil status (%) |  |  |
| Married or in a relationship | 63(58.3) | 297(62.0) |
| Single or widowed | 43(39.8) | 176(36.7) |
| Missing data | 2(1.9) | 6(1.3) |
| Highest education (%) |  |  |
| Primary | 43(39.8) | 105(21.9) |
| Secondary | 39(36.1) | 176(36.7) |
| TertiaryA | 18(16.7) | 137(28.6) |
| TertiaryB | 6(5.6) | 58(12.1) |
| Missing data | 2(1.9) | 3(0.6) |
| Current personal monthly income* (%) |  |  |
| Low | 66(61.1) | 250(52.2) |
| Medium | 36(33.3) | 192(40.1) |
| High | 2(1.9) | 18(3.8) |
| Missing data | 4(3.7) | 19(4.0) |
| BMI^†^ (%) |  |  |
| Underweight | 5(4.6) | 10(2.1) |
| Normal | 27(25.0) | 106(22.1) |
| Overweight | 28(25.9) | 154(32.2) |
| Obesity | 45(41.7) | 198(41.3) |
| Missing data | 3(2.8) | 11(2.3) |
| Smoking status (%) |  |  |
| Never | 27 (25.0%) | 136 (28.4%) |
| Previous | 35 (32.4%) | 180 (37.6%) |
| Current | 43 (39.8%) | 161 (33.6%) |
| Missing data | 3 (2.8%) | 2 (0.4%) |
| CD-RISC-10, mean (SD)^a^ | 20.44(8.90) | 20.77(8.71) |
| GAD-7, mean (SD)^b^ | 11.0 (6.61) | 11.2 (5.68) |
| PHQ-9, mean (SD)^c^ | 13.58(7.84) | 14.35(7.01) |
| PSQI, mean (SD)^d^ | 10.24(4.60) | 10.68(4.19) |

Abbreviations: BMI: Body Mass index. SD: Standard deviation. PSQI: Pittsburgh Sleep Quality Index. PHQ-9: 9-item Patient Health Questionnaire. GAD-7: 7-item Generalized Anxiety Disorder. CD-RISC-10: 10-item version of the Connor-Davidson Resilience Scale.

* Low: ≤$2527/month; Medium: $2528 to $5897/month; High: $5898/month;

† Underweight: BMI< 18.5; Normal weight: BMI 18.5-24.9; Overweight: BMI 25.0-29.9; Obesity: BMI≥ 30.0.

a. Coping ability was assessed with the 10-item version of the Connor-Davidson Resilience Scale (CD-RISC-10)

b. Anxiety symptoms were assessed with the 7-item Generalized Anxiety Disorder (GAD-7).

c. Depressive symptoms were assessed with the 9-item Patient Health Questionnaire (PHQ-9).

d. Sleep symptoms were assessed with the 19-items Pittsburgh Sleep Quality Index (PSQI).

## **Supplementary table 2** Associations between the number of ACEs and the prevalence of A) a severe mental disorder, B) schizophrenia or C) bipolar disorder separately.

|  | All Women, N (%) | Case, N (%) | PR (95% CI) † | PR (95% CI) * |
| --- | --- | --- | --- | --- |
| 1. A severe mental disorder | | | | |
| Total number of ACEs (per ACE) | 29367 (100) | 534 (1.8) | 1.30 (1.27-1.33) | 1.23 (1.20-1.27) |
| By number of ACEs |  |  |  |  |
| 0-2 ACEs | 17354 (59.1) | 155 (29.0) | Ref. | Ref. |
| 3-4 ACEs | 6294 (21.4) | 122 (22.8) | 2.16 (1.73-2.71) | 1.81 (1.44-2.27) |
| ≥5 ACEs | 5719 (19.5) | 257 (48.1) | 4.94 (4.15-5.88) | 3.54 (2.92-4.28) |
| Childhood deprivation |  |  |  |  |
| No | 25472(86.7) | 392 (73.4) | Ref. | Ref. |
| Yes | 3895 (13.3) | 142 (26.6) | 2.38 (2.00-2.83) | 1.76 (1.47-2.10) |
| 1. Schizophrenia | | | | |
| Total number of ACEs (per ACE) | 29367 (100) | 108(0.4) | 1.38 (1.32-1.46) | 1.28 (1.21-1.35) |
| By number of ACEs |  |  |  |  |
| 0-2 ACEs | 17354 (59.1) | 24(0.2) | Ref. | Ref. |
| 3-4 ACEs | 6294 (21.4) | 27(0.5) | 3.11 (1.80-5.35) | 2.43 (1.41-4.17) |
| ≥5 ACEs | 5719 (19.5) | 57(1.0) | 7.11 (4.63-10.9) | 4.40 (2.86-6.79) |
| Childhood deprivation |  |  |  |  |
| No | 25472(86.7) | 75 (0.3) | Ref | Ref |
| Yes | 3895 (13.3) | 33 (0.9) | 2.90 (1.93-4.36) | 1.86 (1.22-2.83) |
| (C) Bipolar disorder | | | | |
| Total number of ACEs (per ACE) | 29367 (100) | 479(1.7) | 1.30 (1.26-1.33) | 1.23 (1.20-1.27) |
| By number of ACEs |  |  |  |  |
| 0-2 ACEs | 17354 (59.1) | 144(0.9) | Ref. | Ref. |
| 3-4 ACEs | 6294 (21.4) | 107(1.8) | 2.04 (1.60-2.60) | 1.72 (1.34-2.21) |
| ≥5 ACEs | 5719 (19.5) | 228(4.2) | 4.73 (3.93-5.68) | 3.45 (2.82-4.23) |
| Childhood deprivation |  |  |  |  |
| No | 25472(86.7) | 350(1.5) | Ref | Ref |
| Yes | 3895 (13.3) | 129(3.5) | 2.42 (2.01-2.92) | 1.83 (1.51-2.21) |

Abbreviations: ACE-IQ: Adverse Childhood Experience International Questionnaire. PR: Prevalence ratio. 95%CI: 95% confidence interval.

†Adjusted for age.

*Additionally adjusted for highest education, civil status, current personal monthly income, smoking status and BMI.

# **Supplementary table 3** Associations between the number of ACEs and the prevalence of severe mental disorder stratified by median age

|  | Women, N (%) | Severe mental disorder, N (%) | PR (95% CI) † | PR (95% CI) * |
| --- | --- | --- | --- | --- |
| <44 years old | | | | |
| Total number of ACEs (per ACE) | 14406 (49.0) | 326 (61.0) | 1.31 (1.28-1.35) | 1.23 (1.19-1.27) |
| Number of ACEs |  |  |  |  |
| 0-2 ACEs | 8399 (58.3) | 87 (26.7) | Ref | Ref |
| 3-4 ACEs | 3061 (21.2) | 75 (23.0) | 2.36 (1.78-3.13) | 1.83 (1.38-2.44) |
| ≥5 ACEs | 2946 (20.5) | 164 (50.3) | 5.38 (4.34-6.67) | 3.40(2.63-4.38) |
| Childhood deprivation |  |  |  |  |
| No | 12466 (86.5) | 229 (70.2) | Ref | Ref |
| Yes | 1940 (13.5) | 97 (29.8) | 2.74 (2.16-3.46) | 1.81 (1.41-2.33) |
| ≥44 years old | | | | |
| Total number of ACEs (per ACE) | 14961 (51.0) | 208 (39.0) | 1.28 (1.23-1.33) | 1.20 (1.15-1.25) |
| Number of ACEs |  |  |  |  |
| 0-2 ACEs | 8955 (59.8) | 68 (32.6) | Ref | Ref |
| 3-4 ACEs | 3233 (21.6) | 47 (22.6) | 1.90 (1.30-2.77) | 1.56 (1.06-2.31) |
| ≥5 ACEs | 2773 (18.6) | 93 (44.8) | 4.31 (3.28-5.66) | 2.97 (2.20-4.02) |
| Childhood deprivation |  |  |  |  |
| No | 13006 (86.9) | 163 (78.4) | Ref | Ref |
| Yes | 1955 (13.1) | 45 (21.6) | 1.83 (1.38-2.43) | 1.36 (1.02-1.81) |

Abbreviations: PR: Prevalence ratio. 95%CI: 95% confidence interval.

† Adjusted for age.

* Additionally adjusted for highest education, civil status, current personal monthly income, smoking status and BMI.

# **Supplementary table 4** Associations between the number of ACEs and the prevalence of severe mental disorder (excluding parental divorce/separation)

|  | **Women, N (%)** | **Severe mental disorder, N (%)** | **PR (95% CI) †** | **PR (95% CI) *** |
| --- | --- | --- | --- | --- |
| **Total number of ACEs (per ACE)** | 29367 (100) | 534 (1.8) | 1.34 (1.31-1.37) | 1.27 (1.23-1.30) |
| **By number of ACEs** |  |  |  |  |
| 0-2 ACEs | 19360 (65.9) | 177 (33.1) | Ref | Ref |
| 3-4 ACEs | 5558 (18.9) | 136 (25.5) | 2.65 (2.10-3.33) | 2.21 (1.74-2.79) |
| ≥5 ACEs | 4449 (15.2) | 221 (41.4) | 5.33 (4.49-6.33) | 3.80 (3.14-4.60) |

Abbreviations: PR: Prevalence ratio. 95%CI: 95% confidence interval.

† Adjusted for age.

* Additionally adjusted for highest education, civil status, current personal monthly income, smoking status and BMI.

# **Supplementary table 5** Associations between the number of ACEs and the prevalence of severe mental disorder (complete cases)

|  | **Women, N (%)** | **Severe mental disorder, N (%)** | **PR (95% CI) †** | **PR (95% CI) *** |
| --- | --- | --- | --- | --- |
| **Total number of ACEs (per ACE)** | 24239 (100) | 397 (1.6) | 1.33 (1.29-1.37) | 1.26 (1.22-1.30) |
| **By number of ACEs** |  |  |  |  |
| 0-2 ACEs | 15194 (62.8) | 122 (30.7) | Ref | Ref |
| 3-4 ACEs | 4936 (20.4) | 88 (22.2) | 2.21 (1.69-2.89) | 1.85 (1.41-2.44) |
| ≥5 ACEs | 4109 (16.8) | 187 (47.1) | 5.58 (4.50-6.90) | 4.01 (3.16-5.09) |

Abbreviations: PR: Prevalence ratio. 95%CI: 95% confidence interval.

† Adjusted for age.

* Additionally adjusted for highest education, civil status, current personal monthly income, smoking status and BMI.

# **Supplementary table 6** Associations between the number of ACEs and the prevalence of severe mental disorder (single imputation using method predictive mean matching to impute ACE-IQ)

|  | All Women, N (%) | Case, N (%) | PR (95% CI) * | PR (95% CI) † |
| --- | --- | --- | --- | --- |
| Total number of ACEs (per ACE) | 29367 (100) | 534 (1.8) | 1.30 (1.27-1.33) | 1.22 (1.19-1.26) |
| By number of ACEs |  |  |  |  |
| 0-2 ACEs | 17206 (58.9) | 152 (28.5) | Ref. | Ref. |
| 3-4 ACEs | 6273 (21.4) | 117 (21.9) | 2.10 (1.66-2.65) | 1.75 (1.38-2.22) |
| ≥5 ACEs | 5888 (19.7) | 265 (49.6) | 5.01 (4.18-5.99) | 3.47 (2.84-4.24) |

Abbreviations: PR: Prevalence ratio. 95%CI: 95% confidence interval.

† Adjusted for age.

* Additionally adjusted for highest education, civil status, current personal monthly income, smoking status and BMI.

# **Supplementary table 7** Associations between the types of ACEs and the prevalence of bipolar disorder or schizophrenia

|  | Bipolar disorder | | Schizophrenia | |
| --- | --- | --- | --- | --- |
|  | **PR (95% CI) †** | **PR (95% CI) *** | **PR (95% CI) †** | **PR (95% CI) *** |
| Abuse  Physical abuse | |  |  |  |
| No | Ref | Ref | Ref | Ref |
| Yes | 2.29 (1.82-2.89) | 1.00 (0.76-1.31) | 2.55 (1.60-4.06) | 0.86(0.49-1.49) |
| Emotional abuse | |  |  |  |
| No | Ref | Ref | Ref | Ref |
| Yes | 2.56 (2.15-3.03) | 1.38 (1.09-1.74) | 3.85 (2.72-5.45) | 2.72 (1.71-4.32) |
| Sexual abuse |  |  |  |  |
| No | Ref | Ref | Ref | Ref |
| Yes | 2.13 (1.78-2.56) | 1.59 (1.31-1.93) | 3.29 (2.21-4.89) | 2.37 (1.52-3.70) |
| Househould dysfunction | |  |  |  |
| Substance abuse | |  |  |  |
| No | Ref | Ref | Ref | Ref |
| Yes | 1.48 (1.23-1.78) | 0.93 (0.76-1.15) | 1.42 (0.99-2.04) | 0.79 (0.51-1.24) |
| Incarcerated household member | |  |  |  |
| No | Ref | Ref | Ref | Ref |
| Yes | 1.47 (1.1-1.98) | 0.89 (0.65-1.22) | 2.35 (1.44-3.81) | 1.50 (0.81-2.79) |
| Mental illness |  |  |  |  |
| No | Ref | Ref | Ref | Ref |
| Yes | 2.67 (2.19-3.26) | 1.96 (1.57-2.43) | 2.40 (1.66-3.45) | 1.49 (1.01-2.19) |
| Family violence | |  |  |  |
| No | Ref | Ref | Ref | Ref |
| Yes | 2.11 (1.8-2.49) | 1.10 (0.89-1.36) | 2.05 (1.41-2.98) | 0.76 (0.46-1.25) |
| Parental separation/divorce | |  |  |  |
| No | Ref | Ref | Ref | Ref |
| Yes | 1.16 (0.97-1.39) | 0.82 (0.68-0.98) | 1.54 (1.02-2.35) | 1.07 (0.68-1.67) |
| Neglect |  |  |  |  |
| Emotional neglect | |  |  |  |
| No | Ref | Ref | Ref | Ref |
| Yes | 2.11 (1.74-2.55) | 1.30 (1.03-1.63) | 2.23 (1.53-3.24) | 1.20 (0.76-1.88) |
| Physical neglect | |  |  |  |
| No | Ref | Ref | Ref | Ref |
| Yes | 2.23 (1.81-2.76) | 1.29 (1.02-1.64) | 1.94(1.24-3.01) | 0.98 (0.58-1.63) |
| Violence |  |  |  |  |
| Bullying |  |  |  |  |
| No | Ref | Ref | Ref | Ref |
| Yes | 2.01 (1.67-2.42) | 1.45 (1.19-1.76) | 2.24 (1.59-3.15) | 1.44 (0.97-2.15) |
| Community violence | |  |  |  |
| No | Ref | Ref | Ref | Ref |
| Yes | 2.20 (1.66-2.91) | 1.22 (0.91-1.64) | 2.44 (1.41-4.2) | 1.17 (0.63-2.18) |
| Collective violence | |  |  |  |
| No | Ref | Ref | Ref | Ref |
| Yes | 2.96 (1.99-4.4) | 1.84 (1.26-2.7) | 4.63 (2.45-8.75) | 2.51 (1.21-5.21) |

Abbreviations: ACE-IQ: Adverse Childhood Experience International Questionnaire. PR: Prevalence ratio. 95%CI: 95% confidence interval.

* Adjusted for age, highest education, civil status, current personal monthly income, smoking status and BMI

† Additionally adjusted for other types of ACEs

# **Supplementary** **table 8** Linear regression to assess the association between types of ACE and psychological functioning among women with severe psychiatric disorders (n= 534)

|  | Anxiety^a^ | Depression^b^ | Sleep^c^ | Coping ability^d^ |
| --- | --- | --- | --- | --- |
| Abuse |  |  |  |  |
| Physical abuse | **β(95% CI)*** | **β(95% CI)*** | **β(95% CI)*** | **β(95% CI)*** |
| No | Ref | Ref | Ref | Ref |
| Yes | 0.35(-1.1,1.8) | 0.41(-1.39,2.2) | 0.72(-0.19,1.64) | 1.73(0.09,3.38) |
| Emotional abuse |  |  |  |  |
| No |  |  |  |  |
| Yes | 1.29(0.6,1.98) | 1.74(0.78,2.71) | 0.65(-0.31,1.61) | 1.16(-0.7,3.03) |
| Sexual abuse |  |  |  |  |
| No | Ref | Ref | Ref | Ref |
| Yes | 1.35(0.99,1.70) | 1.7(1.41,2.00) | 1.17(0.77,1.57) | -0.51(-1.57,0.54) |
| Househould dysfunction |  |  |  |  |
| Substance abuse |  |  |  |  |
| No | Ref | Ref | Ref | Ref |
| Yes | 0.43(0.09,0.76) | 0.71(0.05,1.38) | 0.16(-0.1,0.42) | -0.25(-0.8,0.3) |
| Incarcerated household member | |  |  |  |
| No | Ref | Ref | Ref | Ref |
| Yes | -0.8(-2.53,0.93) | -1.29(-3.16,0.59) | -0.82(-1.66,0.02) | 1.41(-0.6,3.42) |
| Mental illness |  |  |  |  |
| No | Ref | Ref | Ref | Ref |
| Yes | 0.56(0.01,1.12) | 1.12(0.61,1.63) | 0.3(-0.16,0.76) | 0.33(-1.19,1.84) |
| Family violence |  |  |  |  |
| No | Ref | Ref | Ref | Ref |
| Yes | 1.61(0.68,2.53) | 1.83(0.12,3.54) | 0.42(-0.52,1.36) | -0.97(-3.18,1.24) |
| Parental separation/divorce |  |  |  |  |
| No | Ref | Ref | Ref | Ref |
| Yes | 0.02(-1.07,1.11) | -0.2(-1.62,1.21) | -0.37(-0.63,-0.11) | -0.67(-1.48,0.15) |
| Neglect |  |  |  |  |
| Emotional neglect |  |  |  |  |
| No | Ref | Ref | Ref | Ref |
| Yes | 1.69(1.27,2.12) | 2.45(1.88,3.02) | 1.25(0.89,1.6) | -1.59(-3.03,-0.15) |
| Physical neglect |  |  |  |  |
| No | Ref | Ref | Ref | Ref |
| Yes | 1.56(1.29,1.83) | 1.89(0.59,3.2) | 0.82(0.1,1.55) | -0.87(-2.17,0.44) |
| Violence |  |  |  |  |
| Bullying |  |  |  |  |
| No | Ref | Ref | Ref | Ref |
| Yes | 1.32(0.57,2.07) | 1.49(0.84,2.14) | 0.48(-0.41,1.37) | -0.26(-1.09,0.56) |
| Community violence |  |  |  |  |
| No | Ref | Ref | Ref | Ref |
| Yes | 1.95(0.84,3.06) | 1.83(0.9,2.76) | 1.57(0.73,2.42) | 0.11(-0.67,0.89) |
| Collective violence |  |  |  |  |
| No | Ref | Ref | Ref | Ref |
| Yes | 1.43(0.78,2.07) | 1.04(-1.47,3.55) | 1.07(0.53,1.62) | 1.34(0.06,2.63) |

Abbreviations: BMI: Body Mass index. PSQI: Pittsburgh Sleep Quality Index. PHQ-9: 9-item Patient Health Questionnaire. GAD-7: 7-item Generalized Anxiety Disorder. CD-RISC-10: 10-item version of the Connor-Davidson Resilience Scale.

* Adjusted for age, highest education, civil status, current personal monthly income, smoking status and BMI

a. Anxiety symptoms were assessed with the 7-item Generalized Anxiety Disorder (GAD-7), with higher scores indicating greater symptom severity.

b. Depressive symptoms were assessed with the 9-item Patient Health Questionnaire (PHQ)-9, with higher scores indicating greater symptom severity.

c. Sleep symptoms were assessed with the 19-items Pittsburgh Sleep Quality Index (PSQI) , with higher scores indicating greater symptom severity.

d. Coping ability was assessed with the 10-item Connor-Davidson Resilience scale (CD-RISC-10), with lower scores indicating worse resilience.

# **Supplementary table 9** Poisson regression to assess the association between ACEs and psychological functioning among women with severe mental disorder (n= 534)

|  | **Women** | **Anxiety^a^** | **Depression^b^** | **Sleep^c^** |
| --- | --- | --- | --- | --- |
|  |  | **PR(95% CI)*** | **PR(95% CI)*** | **PR(95% CI)*** |
| **Number of ACEs** |  |  |  |  |
| 0-2 ACEs | 155 | Ref | Ref | Ref |
| 3-4 ACEs | 122 | 1.02(0.81-1.29) | 1.09(0.96-1.25) | 0.99(0.95-1.02) |
| ≥ 5 ACEs | 257 | 1.29(1.08-1.55) | 1.27(1.18-1.36) | 1.10(1.00-1.21) |
| **Childhood deprivation** |  |  |  |  |
| No | 392 | Ref | Ref | Ref |
| Yes | 144 | 1.09(1.04-1.13) | 1.09(1.06-1.12) | 1.03(0.96-1.10) |

Abbreviations: BMI: Body Mass index. PSQI: Pittsburgh Sleep Quality Index. PHQ-9: 9-item Patient Health Questionnaire. GAD-7: 7-item Generalized Anxiety Disorder. CD-RISC-10: 10-item version of the Connor-Davidson Resilience Scale.

* Adjusted for age, highest education, civil status, current personal monthly income, smoking status and BMI

a. Anxiety symptoms were assessed with the 7-item Generalized Anxiety Disorder (GAD-7), with a score of ≥10 as the cutoff for a probable case

b. Depressive symptoms were assessed with the 9-item Patient Health Questionnaire (PHQ)-9, with a score of ≥10 as the cutoff for a probable case

c. Sleep symptoms were assessed with the 19-items Pittsburgh Sleep Quality Index (PSQI) , with a score of ≥5 as the cutoff for a probable case


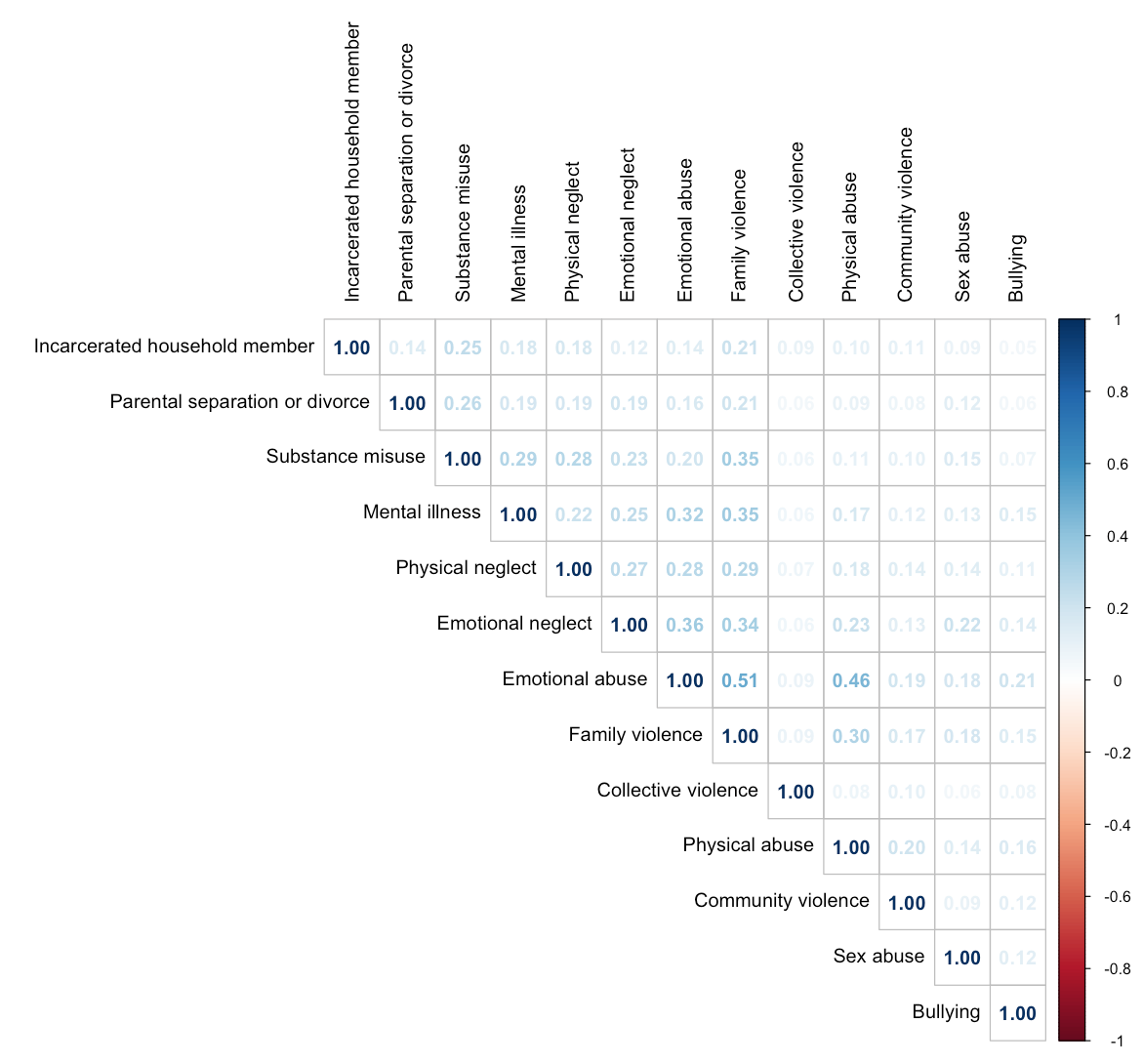


# **Supplementary Figure 1** Rank order correlations for ACE subtypes
